# Fluid intake, fluid output or fluid balance, which one matters in ARDS

**DOI:** 10.1186/s13054-022-04188-6

**Published:** 2022-11-14

**Authors:** Yanfei Shen, Guolong Cai, Jing Yan

**Affiliations:** Department of Intensive Care, Zhejiang Hospital, No. 1229, Gudun Road, Hangzhou, 310013 Zhejiang People’s Republic of China

To the editor,

In a recent trial including 650 COVID-19 patients with acute respiratory distress syndrome (ARDS) [1], Dr. Ahuja et al. reported a linear association between cumulative fluid balance and successful liberation from invasive ventilation in the restricted cubic spline function models. This study is well designed, and the conclusions are clear. We want to add some comments.

The volume of fluid balance is calculated as fluid intake—fluid output. Although dozens of studies have reported that increased fluid balance volume is associated with poor prognosis in various diseases, it remains unclear which factor really matters with the prognosis: Fluid input? Fluid output or fluid balance? For instance, in the current study, the fluid intake volume within three days was quite close between the lower and higher tertiles, and the difference in fluid balance was mainly caused by the difference in urine output. However, is urine output or fluid balance the key factor in this relationship?

In clinical practice, fluid intake, output and fluid balance have complex interactions. Based on our previous findings, we suggest that the fluid accumulation index, which is derived from both fluid balance and fluid intake (fluid balance/fluid intake = 1 − fluid output/fluid intake), may play a vital role in the relationship with prognosis. For instance, studies have reported that patients with severe capillary leak may have a worse prognosis [2, 3]. However, as shown in Fig. 1, with different fluid intake and capillary leak severity, the fluid balance is the same in patients A and B, while the fluid accumulation index is different (0.4 vs. 0.8). In addition, our previous finding [4] also revealed a complete mediation relationship within fluid balance, fluid balance/fluid intake and mortality in sepsis, which suggested that the association between fluid balance and mortality is completely mediated by fluid balance/fluid intake ratio [5]. Therefore, compared to fluid balance, fluid intake or fluid output alone, the fluid accumulation index (fluid balance/fluid intake) can more accurately reflect the ability to excrete excessive fluid under different fluid loads and predict the prognosis.

**Fig. 1:**
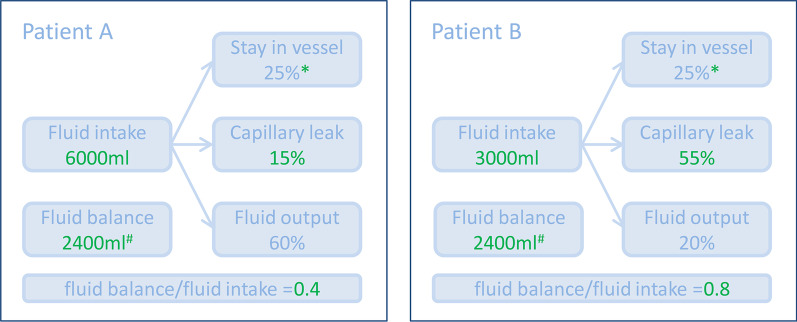
Distribution of intravenous crystalloid fluid in two hypothetic patients. Patient A: fluid intake 6000 ml, 15% of which goes into interstitial issue due to capillary leak and 60% was fluid output. Patient B: fluid intake 3000 ml, 55% of which goes into interstitial issue due to capillary leak and 20% was fluid output. Note: *25% is assumed according to one study (PMID: 34040918); # Fluid balance = fluid intake – fluid output

Finally, Dr. Ahuja et al.’s study added important information to the current knowledge of COVID-19, and their findings are highly appreciated. We hope our hypothesis will be helpful for further research.

## Data Availability

Not applicable.
