# Supplementary material for: Fluid intake, fluid output or fluid balance, which one matters in ARDS
Source: Crit Care. 2022 Nov 14;26:352. doi: 10.1186/s13054-022-04188-6 (PMC9662122; doi:10.1186/s13054-022-04188-6)
Supplement: Related texts

# Reply: Fluid intake, fluid output or fluid balance: Which one does not matter in ARDS?

**Authors:** Sanchit Ahuja, Harm-Jan de Grooth, Marcus J. Schultz

**Affiliations:** 2 grid.239578.2 0000 0001 0675 4725 Department of Cardiothoracic Anesthesiology, Cleveland Clinic, Cleveland, OH USA; 3 grid.239578.2 0000 0001 0675 4725 Outcomes Research Consortium, Cleveland Clinic, Cleveland, OH USA; 4 grid.16872.3a 0000 0004 0435 165X Department of Intensive Care, Amsterdam UMC, location VUmc, Amsterdam, The Netherlands; 5 grid.509540.d 0000 0004 6880 3010 Department of Intensive Care, C3–415, Amsterdam UMC, location AMC, Meibergdreef 9, 1105 AZ Amsterdam, The Netherlands; 6 grid.10223.32 0000 0004 1937 0490 Mahidol Oxford Tropical Medicine Research Unit (MORU), Mahidol University, Bangkok, Thailand; 7 grid.4991.5 0000 0004 1936 8948 Nuffield Department of Medicine, University of Oxford, Oxford, UK

In your journal, we recently showed that a higher cumulative fluid balance has an association with a longer duration of ventilation in patients with acute respiratory distress syndrome (ARDS) due to coronavirus disease 2019 (COVID-19), suggesting that restricted fluid management in these patients may be beneficial [1].

Shen et al. correctly mention that fluid intake, fluid output and fluid balance have complex interactions, and that a ‘fluid accumulation index,’ an index derived from both the fluid balance and the fluid intake, may play a vital role in this association [4].

To test this hypothesis, we reanalyzed the database of ‘Practice of Ventilation in COVID–19 patients’ (PRoVENT–COVID), the study we used to test our original hypothesis [6]. Here, we present a sensitivity analysis that modeled the successful liberation of ventilation as a function of the fluid accumulation index in the Cox regression model after adjusting for potential confounders defined in our study. We also adjusted for the ‘fluid accumulation index’ and refitted our main regression model, i.e., cumulative fluid balance on the hazard of successful liberation from invasive ventilation.

The fluid accumulation index was not associated with successful liberation of ventilation, even after adjusting for potential confounders (*P* = 0.2853) (Fig. 2A). The association between cumulative fluid balance at day 3 and successful liberation of ventilation remained consistent by adjusting for the fluid accumulation index (*P* = 0.0068) (Fig. 2B).

**Fig. 2:** Marginal effect plots. **A** Association between day 3 fluid accumulation index and successful liberation of ventilation, adjusted for the potential confounder variables described in our study. **B** Association between cumulative fluid balance at day 3 and our primary outcome, adjusted for day 3 fluid accumulation index and the potential confounder variables described in our study

Based on these findings, we conclude that the fluid accumulation index has no added value in patients with ARDS due to COVID-19.
